# The effect of frequent plasmapheresis in a patient with anti-glomerular basement membrane antibody diseases with severe renal dysfunction: a case report and literature review

**DOI:** 10.3389/fimmu.2026.1825085

**Published:** 2026-06-12

**Authors:** Huimin Tian, Qian Ren, Hongyu Qiu, Shenju Gou

**Affiliations:** Department of Nephrology and Institute of Kidney Diseases, West China Hospital, Sichuan University, Chengdu, China

**Keywords:** anti-glomerular basement membrane antibody, case report, crescentic glomerulonephritis, plasmapheresis, rapidly progressive glomerulonephritis

## Abstract

Anti-glomerular basement membrane antibody (anti-GBM) disease is a rare yet aggressive autoimmune disorder that affects the kidneys and lungs. Patients with renal involvement often progressed to death or end-stage renal disease. Treatment with plasmapheresis, along with cyclophosphamide and corticosteroids, has been suggested to improve renal and patient survival; however, it showed little benefit in patients who presented with severe disease. We herein report a critically ill case with anuria, creatinine value of 752 μmol/L, anti-GBM antibody titer of 92.54 RU/mL, and 96.7% fibro-cellular crescent formation at renal biopsy. The patient was diagnosed with anti-GBM disease and treated with plasmapheresis and immunosuppressive therapy. Plasmapheresis was not ceased until the total clearance of pathogenic autoantibodies. The patient’s renal function was successfully rescued and maintained normal during the 5-year follow-up. Our experience implicated that early and persistent plasmapheresis could rescue the kidney function of anti-GBM patients who have extremely poor prognosis.

## Introduction

Anti-glomerular basement membrane (anti-GBM) antibody disease is an autoimmune-mediated disorder with an estimated incidence ratio of one to two cases per million population per year. It is characterized by the development of pathogenic autoantibodies targeting autoantigens expressed in the basement membranes of the kidneys and lungs. Patients often presented with rapidly progressive glomerulonephritis (RPGN), while an estimated proportion of 40%–60% had concurrent alveolar hemorrhage (1). Patients of severe anti-GBM diseases had poor prognosis, and a recent investigation showed that the 5-year kidney survival rate of anti-GBM GN was 34% (2). The recommended treatment of plasma exchange, cyclophosphamide, and corticosteroids, combined with early diagnosis, is commonly accepted as it could improve patient outcomes. However, for patients with severe conditions, such as dialysis dependence at presentation or a high proportion of glomerular crescents at biopsy, such therapeutic strategy seemed to be of little help (2, 3). Whether there is an opportunity of recovery for these patients and how to treat them in a more effective way remain unclear. We herein report the successful rescue of renal function in a patient with anti-GBM disease with extremely poor initial kidney function. Besides regular immunosuppressive therapy, the patient was treated with persistent plasmapheresis until the removal of anti-GBM antibodies. Her renal function was recovered after 25 rounds of plasmapheresis, and this was remained normal during the follow-up period.

## Case presentation

A previously healthy 26-year-old woman was admitted to our hospital with complaints of recurrent fever, nausea, vomiting, and renal dysfunction. Nineteen days ago, she developed chills, fever, fatigue, and poor appetite after catching a cold, with a maximum body temperature of 39.3 °C. Ten days ago, besides the persistent primary symptoms, additional symptoms, including upper abdominal pain, diluted stool, nausea, vomiting, and a hacking cough, appeared. She was given ciprofloxacin in a local hospital, but it did not show any help after 3 days of treatment. Then, she noticed mild edema of her lower limbs, so a further check-up was conducted at the outpatient clinic of her local hospital. The urinary analysis showed 37 red blood cells (RBCs) per high-power field (HP) and 1+ of proteinuria. Two days later, dysuria, oliguria, and dark yellow urine were noticed. The serum creatinine revealed by biochemical tests was 752 μmol/L. The blood routine test showed a low hemoglobin concentration of 84 g/L and high white blood cell (WBC) count of 15*10^9^/L, with 88.2% neutrophilic segmented granulocyte. Immunoassay detected positivity of cytoplasmic anti-neutrophil cytoplasmic antibodies cANCA 1:100, PR3-ANCA antibody (++), and anti-GBM antibodies. Bilaterally enlarged kidneys with slightly enhanced essence echoes were revealed by color Doppler ultrasound, and a chest CT scan found a small bilateral pleural effusion with a few strip-shaped shadows at the inferior left lobe. She was diagnosed with anti-GBM glomerulonephritis and a pulmonary infection. Plasmapheresis was initiated immediately every other day, combined with an injection of methylprednisolone (1 g) and gamma globulin (20 g) daily for three consecutive days. After two sessions of plasmapheresis, the body temperature went back to normal, and there was no more abdominal pain and diarrhea. However, the symptoms of hacking cough, nausea, vomiting, dysuria, and oliguria still existed. The daily urine volume dropped to 100~200 mL, and symptoms of shortness of breath and inability to lie flat at night appeared. The patient has no history of chronic diseases, hepatitis, tuberculosis, or allergies. The patient denies any history of surgery. There are no risk factors and no known autoimmune diseases in the family history. Prior to the onset of the acute illness, the patient was in good health. Therefore, she was transferred to our center for further treatment.

**Figure 1:**
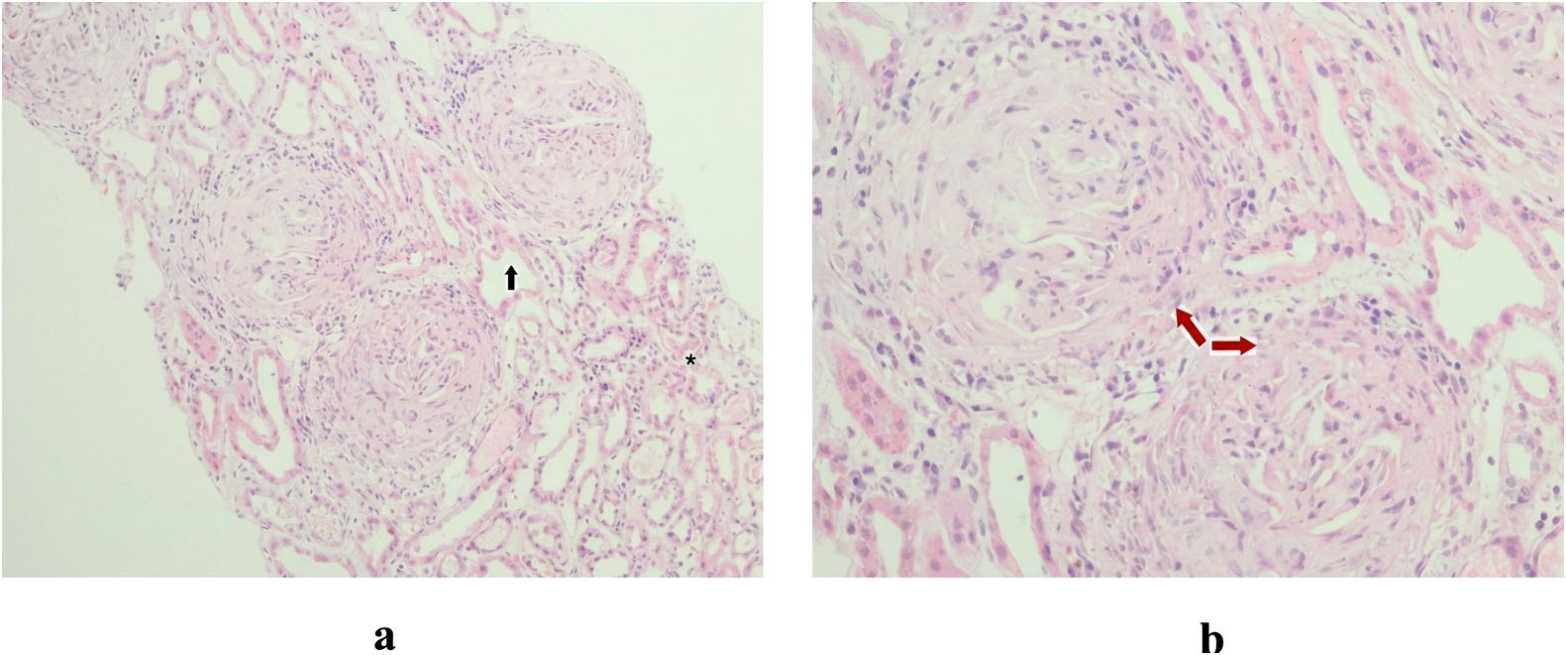
Renal biopsy findings: **(a)** light microscopy (HE stains, ×200) revealed flattened tubular epithelial cells with cellular swelling and cytoplasmic vacuolization (black arrow) as well as cast formation (asterisk). **(b)** Representative glomerulus (HE stains, ×400) containing fibrocellular crescents is shown (red arrow).

On admission, her body temperature was 35.8 °C and the blood pressure was 112/67 mmHg. The laboratory tests showed aggravated anemia with hemoglobin decreased to 72 g/L. The WBC count was 20.86*10^9^/L, and 97.1% neutrophilic segmented granulocytes. The serum creatinine was 497.5 μmol/L and the serum albumin and Ca^2+^ were 23.9 g/L and 1.97 mmol/L, respectively. The ANCA test was negative, but the anti-GBM antibody was persistently positive. The level of anti-GBM antibody was 92.54 RU/mL (normal range <20RU/mL). Doppler ultrasound measured the right kidney at 11.5 cm × 5.9 cm × 5.5 cm and the left kidney at 12.3 cm × 5.3 cm × 4.9 cm. Because of her severe condition, a percutaneous renal biopsy was not performed until the 13th day of hospitalization (**Figure 1**). Among 32 qualified glomeruli, 31 glomeruli revealed fibrocellular crescents and circumferential crescents. The involved glomeruli were shrunk, and only one glomeruli was nearly normal under light microscopy. No obvious atrophy was noticed in the tubulointerstitial compartment. The tubular changes include flattened or missing brush border, hydropical epithelium, vacuolar degeneration, and erythrocyte or protein cast formation. Lymphocytes, monocytes, and plasma cells could be found sporadically in the interstitium, and there was no obvious vascular lesion. Immunofluorescence testing showed linear deposition of IgG, C3, and C1q along the GBMs. With these pathological features, she was diagnosed with crescentic glomerulonephritis (type I).

**Figure 2:**
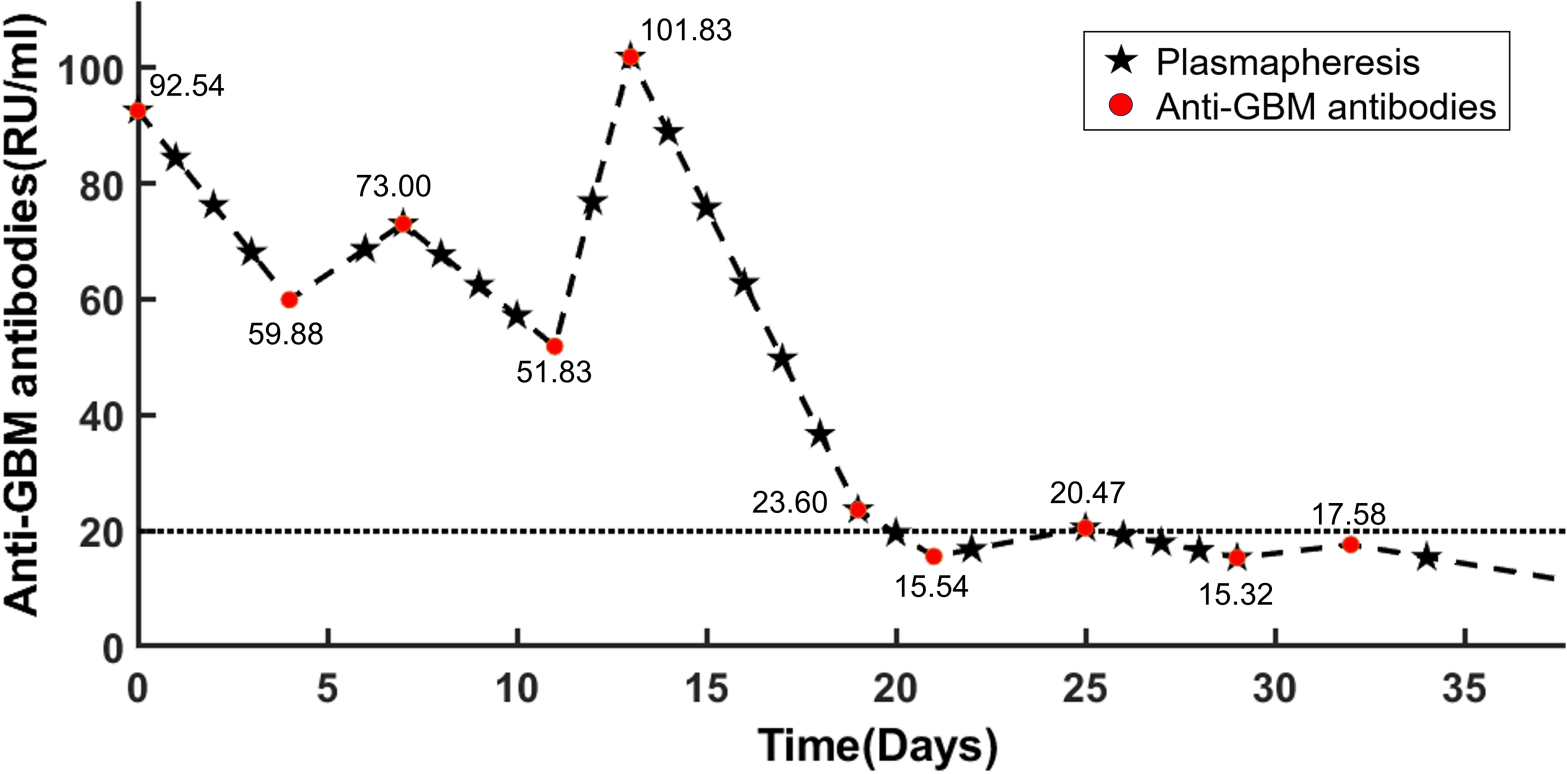
Changes in antibody titers following plasma exchange therapy.

**Figure 3:**
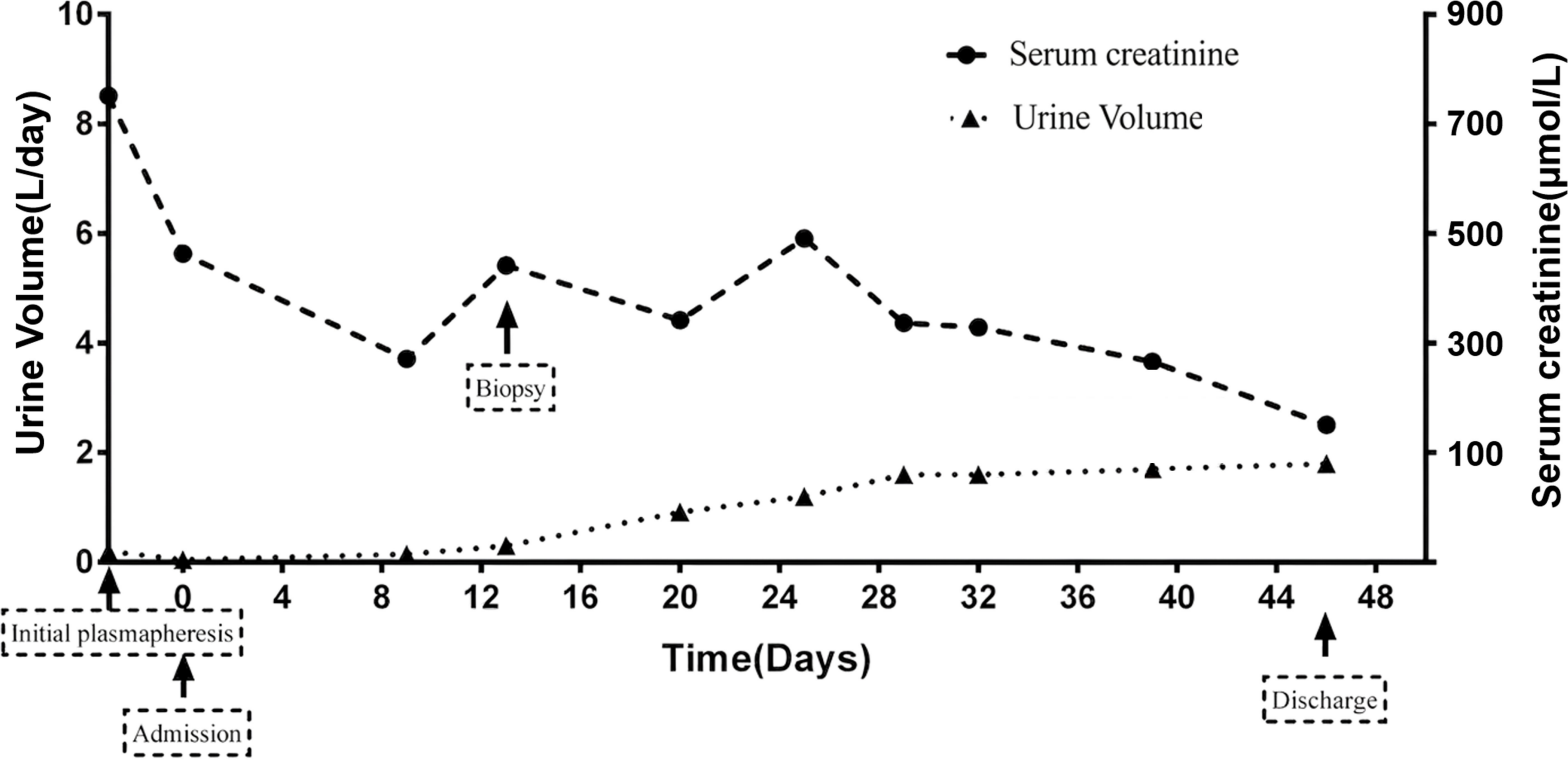
Changes in urine output and creatinine levels.

Intermittent hemodialysis every other day was continued, and therapeutic plasma exchange was repeated. After 14 sessions of plasmapheresis, the anti-GBM antibody was still positive and the serum creatine concentration was 390.7 μmol/L. Plasmapheresis was not stopped until anti-GBM antibodies were no longer detectable, and it was followed by regular hemodialysis. In total, she underwent 25 rounds of plasmapheresis, including 10 sessions of traditional plasma exchanges with 1,500–3,000 mL plasma and 15 sessions of double filtration plasmapheresis with 600~800 mL plasma, all of which used fresh frozen plasma combined with regular plasma as the replacement volume, and the blood flow rate was 120–150 mL/min, with a replacement rate of 20–30 mL/h (**Figure 2**). During the 25 sessions of plasmapheresis, the patient’s coagulation profile, including PT, APTT, and fibrinogen levels, was closely monitored. Although a transient decrease in fibrinogen was observed following the frequent plasmapheresis, the levels remained within an acceptable clinical range. No bleeding events (such as alveolar hemorrhage) or thrombotic complications were recorded throughout the treatment period. No new-onset severe infections or sepsis occurred during the treatment course. In addition, the patient did not encounter any vascular complications, such as thrombosis or catheter-related infections. The urine volume gradually returned to normal as 1,500–2,000 mL (**Figure 3**). The serum creatine decreased to 222.5 μmol/L, and the serum albumin increased to 35 g/L (**Table 1**). Hemodialysis was thus stopped and she was discharged with continued outpatient therapy, including methylprednisolone at a dosage of 40 mg per day, which was gradually tapered over the subsequent months. Concurrently, intravenous cyclophosphamide pulses (600 mg per month) were administered, until the cumulative dose reaches 6.0 grams. Five months later, her serum creatinine decreased to 148 μmol/L. Despite sustained proteinuria (+) and microscopic hematuria (3–10 RBC/HP), she presented generally well with 2,000–2,500 mL urine volume and 130–150 μmol/L serum creatinine concentration after 1 year. At follow-up after 5 years, she showed a nearly normal kidney function and a normal urinalysis. The persistent negative anti-GBM antibodies and ANCA were tested every 3–6 months during the follow-up periods. Her most recent tests showed a serum creatinine level of 115.0 μmol/L, negative proteinuria, and two RBCs per high-power field.

**Table 1:** Laboratory data on admission and at discharge in our hospital.

Urinalysis and coagulation	Admission	Discharge	Blood routine examination	Admission	Discharge	Blood biochemistry	Admission	Discharge	Serology	Admission	Discharge
Pro	0.7 (1+) g/L	1.0 (2+) g/L	WBC	20.86*10^9^/L	7.94*10^9^/L	TBIL	4.2 umol/L	4.2 umol/L	ANA	(-)	(-)
RBC	37/HP	8/HP	RBC	2.53*10^12^/L	2.81*10^12^/L	AST	36 IU/L	19IU/L	ANCA-MPO	<1.0	<1.0
WBC	8/HP	2/HP	PLT	274*10^9^/L	129*10^9^/L	ALT	19 IU/L	20 IU/L	ANCA-PR3	<1.0	<1.0
			NET%	97.1%	40.75%	TP	50.6 g/L	59.4 g/L	Anti-GBM	92.54 RU/mL	NA
APTT	45.4	25.2	Hb	72 g/L	85g/L	ALB	23.9 g/L	33.5 g/L	C3	0.5240 g/L	NA
TT	28.0	20.5	Hct	0.22 L/L	0.25 L/L	GLB	26.7 g/L	25.9 g/L	C4	0.1130 g/L	NA
INR	1.27	1.17	MCV	85.8 fL	89 fL	Glu:	9.24 mmol/L	4.37 mmol/L	CD3	60.9%	NA
PT	14.2	13.0	MCH	28.5 pg	30.6 pg	BUN:	17.10 mmol/L	21.05 mmol/L	CD4	28.6%	NA
FIB	1.51	1.90	MCHC	332 g/L	344 g/L	Crea:	497.5 umol/L	222.5 umol/L			
						eGFR:	9.72 mL/min/1.73 m^2^	20.95 mL/min/1.73 m^2^			
						Cys-c:	5.22 mg/L	3.87 mg/L			
						Uric:	348.0 umol/L	387 umol/L			
						GGT:	51 IU/L	51 IU/L			
						CK:	21 IU/L	21 IU/L			
						TG:	0.84 mmol/L	1.58 mmol/L			
						Chol:	2.64 mmol/L	5.21 mmol/L			
						LDL-C:	0.97 mmol/L	2.98 mmol/L			
						HDL:	1.24 mmol/L	1.43 mmol/L			

## Discussion

The key to anti-GBM disease is the appearance of circulating autoantibodies targeting type IV collagen in the glomerular or alveolar basement membrane. These autoantibodies, typically belonging to the IgG type and sometimes IgA or IgM, bind rapidly and tightly to the NC1 domain of the α3 chain, activating the complement system, recruiting and binding to neutrophils and macrophages that could release lysozyme and cytokines, and finally contributing to the rupture of GBM and generation of local inflammation (4, 5). Patients with renal involvement often manifest as RPGN that could progress rapidly to severe oliguria or anuria and end-stage diseases. This patient presented with prominent systemic symptoms and advanced kidney dysfunction. Meanwhile, the results from serology and pathology found increased anti-GBM antibodies and deposition of IgG and C3. A diagnosis of anti-GBM diseases was made. It was noteworthy that the patient had a transient positivity for ANCA. The appearance of ANCAs might explain her prominent systemic symptoms but could not help reach the diagnosis of ANCA-associated vasculitis as they were undetectable thereafter. As a matter of fact, it was estimated that nearly 33% anti-GBM cases had coexistence of ANCA, and this antigenically distinct antibody type was more associated with the relapse of diseases instead of early morbidity and mortality in double-positive cases (6). Although the ANCA of this patient turned negative relatively quickly, the complexity of patients with dual positivity (both ANCA- and anti-GBM-positive) cannot be ignored. A recent large retrospective study pointed out that although patients with dual positivity exhibit symptoms similar to typical anti-GBM disease during the acute phase, their long-term management should be more inclined toward the follow-up model for ANCA-associated vasculitis, as these patients have a higher risk of relapse, which is significantly different from the characteristic of “usually non-relapsing” in patients with single positive anti-GBM (7).

Given that circulating anti-GBM autoantibodies are the major factors causing irreversible kidney damage, rapid removal of these pathogenic autoantibodies is crucial. In this context, plasma exchange has emerged as a critical mechanical intervention. The primary physiological benefit of plasma exchange lies in its ability to rapidly reduce the burden of circulating anti-GBM antibodies and inflammatory mediators, thereby halting the further destruction of the basement membrane (8). However, a key biological challenge in anti-GBM disease is the phenomenon of “antibody rebound”. Because a significant portion of pathogenic IgG is sequestered in the extravascular space and bound to the glomerular basement membrane, discontinuation of short-term plasma exchange often leads to rapid redistribution and rebound of serum antibody titer (3). To overcome this issue, plasmapheresis must be used in combination with potent immunosuppressants (such as cyclophosphamide and corticosteroids). Plasmapheresis clears existing antibodies, while immunosuppression is essential to inhibit the *de novo* synthesis of autoantibodies by B cells and plasma cells, ultimately ensuring sustained serological and clinical remission (9). Patients with severe anti-GBM disease, if untreated, do not recover their renal function and are at high risk of death (10). The employment of plasmapheresis, cyclophosphamide, and corticosteroids, which aims to rapidly remove pathogenic autoantibodies, prevent further autoantibody production, and ameliorate tissue inflammation, has revolutionized outcomes. According to recent investigations, the overall 1-year patient survival of anti-GBM was 70%–80%, and it does not show any difference among races. The kidney survival rate at 1 year, however, ranges between 20% and 40% from center to center. This discrepancy might be attributed to the fact that patients in different studies had varying disease severity (10, 11). The current consensus was that the combined therapy was effective in patients that did not show very advanced kidney failure at presentation. In patients with less than 500 μmol/L creatinine value, the 1-year patient and renal survival could reach up to 100% and 95%, respectively (12). For those who presented with very advanced kidney failure, the effectiveness of intensive treatment remains controversial. Generally, oliguria at diagnosis, over 500 μmol/L initial creatinine concentration, high crescent formation at biopsy, and dialysis requirement at presentation were predictors of poor outcomes. In one retrospective study, oliguria presented as the strongest predictor of patient and renal survival. In the same study, no patient with oliguria recovered their renal function (10). Another study showed that in patients with a creatinine concentration of 500 μmol/L or more, the patient and renal survival were 83% and 82% at 1 year, and in patients with initial dialysis dependence, the values were 65% and 8%, respectively (12). A recent investigation in a large, international cohort of patients also found that patients who were dialysis dependent at presentation did not benefit from intensive treatments. Besides this, they also found that those who had 100% cellular crescents at biopsy never recovered their kidney function and were not able to discontinue dialysis (2). In our case, the patient had a urine volume of 100–200 mL/day, a presenting creatinine value of 752 μmol/L, a high fibro-cellular crescent portion of 97%, and dialysis requirement at presentation. All of these strongly indicated that her prognosis was not optimistic, and there existed much difficulty to rescue her dysfunctional kidneys. Nevertheless, we initiated early plasmapheresis along with immunosuppressive therapy and continued plasmapheresis until the complete absence of anti-GBM antibodies. Such therapeutic strategy successfully freed her from dialysis and restored her kidney function. This suggested that early and aggressive therapy might also be meaningful for those with very advanced kidney failure.

It has been commonly accepted that early diagnosis and early induction of plasmapheresis, together with immunosuppression, are crucial for achieving optimum overall and renal outcomes. The question of how many rounds of plasmapheresis should be performed and when therapy should be terminated has not yet been well answered. A new statement by the American Society for Apheresis, however, noted that the minimum course of plasmapheresis should be 10–20 days, and the presence or absence of antibodies should not be considered in the decision to terminate therapy (13). Nevertheless, the titers of anti-GBM antibodies should be closely monitored as they reflect the progression of anti-GBM GN1. As for our patient, we continued plasmapheresis with periodical checking of antibody levels. Termination of therapy was called when there were no more detectable circulating antibodies. In total, she received 25 rounds of plasmapheresis and finally got complete remission, without any dialysis dependence or relapse of disease. Persistent plasmapheresis might lead to side effects such as hemorrhage, allergic reactions, and increased risk of infection. For our patient, however, none of these mentioned symptoms were noticed, implying the feasibility of our strategy. The management of the present case reflects an evolution in the therapeutic philosophy for anti-GBM disease. The prevailing KDIGO 2012 guidelines recommended plasmapheresis for 14 days or until the antibodies were undetectable (14). In the present case, the favorable outcome was achieved after GBM antibodies successfully turned negative with long-term plasma exchange treatment of 25 sessions, which far exceeded 14 times. The present case supports the adjustments made by KDIGO in 2021, which set the goal of antibody clearance as the sole target for plasma exchange, without a fixed treatment duration, although the reported average antibody clearance time was 8–14 days (15). By demonstrating sustained renal recovery (serum creatinine <115.0 µmol/L) at a 5-year follow-up, our report provides robust evidence that the “until undetectable” strategy should be relentlessly pursued, even in clinically dismal presentations. This adds a crucial layer of evidence to the 2021 KDIGO framework, suggesting that the limit of “therapeutic futility” may need to be re-evaluated in the era of individualized, antibody-guided therapy. This is in consistence with a currently reported case where treatment with plasmapheresis until the disappearance of anti-GBM antibodies improved the prognosis of a patient with advanced kidney dysfunction (16). What is different was that in the reported case, the 100% crescents were all cellular crescents and represented a reversible renal damage. The present case, however, showed 97% fibro-cellular crescents or circumferential crescent formation. The successful treatment of our patient implies that persistent plasmapheresis with immunosuppressive therapies might still be effective in cases with advanced pathological injuries.

In summary, we reported a case of anti-GBM disease that presented with advanced kidney dysfunction and was successfully treated by persistent plasmapheresis and immunosuppressive therapies. The present case firstly indicated that patients with predictors of poor outcomes, such as anuria, a creatinine value over 500 µmol/L, high fibrocyte crescent portion, and initial dialysis requirement, still have a chance to get total recovery. Moreover, early intensive treatments might contribute to good prognosis and should be initiated without delay if the diagnosis was highly suspected, even if there was a lack of pathological confirmation. Lastly, extending the duration of plasmapheresis until the disappearance of autoantibodies might be helpful for the recovery of kidney function. The effectiveness and standard protocols of plasmapheresis in treating anti-GBM disease still need further studies.

## Data Availability

The original contributions presented in the study are included in the article/supplementary material. Further inquiries can be directed to the corresponding authors.

## References

[1] McAdoo SP Pusey CD . Anti-glomerular basement membrane disease. Clin J Am Soc Nephrol. (2017) 12:1162–72. doi: 10.1007/978-0-387-84828-0_53 28515156 PMC5498345

[2] van Daalen EE Jennette JC McAdoo SP Pusey CD Alba MA Poulton CJ . Predicting outcome in patients with anti-GBM glomerulonephritis. Clin J Am Soc Nephrol. (2018) 13:63–72. doi: 10.2215/cjn.04290417 29162595 PMC5753308

[3] McAdoo SP Pusey CD . Anti-glomerular basement membrane disease-treatment standard. Nephrol Dial Transplant. (2025) 41:42–54. doi: 10.1093/ndt/gfaf190 40973182 PMC12722177

[4] Lahmer T Heemann U . Anti-glomerular basement membrane antibody disease: a rare autoimmune disorder affecting the kidney and the lung. Autoimmun Rev. (2012) 12:169–73. doi: 10.1016/j.autrev.2012.04.002 22546293

[5] Kuang H Wan HS Cui Z Zhao MH Jia XY . Elevated serum soluble α3(IV)NC1 correlates with kidney injury and worse outcome in patients with anti-glomerular basement membrane disease. Front Immunol. (2026) 17:1728059. doi: 10.3389/fimmu.2026.1728059 41798942 PMC12963254

[6] McAdoo SP Tanna A Hrušková Z Holm L Weiner M Arulkumaran N . Patients double-seropositive for ANCA and anti-GBM antibodies have varied renal survival, frequency of relapse, and outcomes compared to single-seropositive patients. Kidney Int. (2017) 92:693–702. doi: 10.1016/j.kint.2017.03.014 28506760 PMC5567410

[7] Philip R Dumont A Le Mauff B Martinet M Martin Silva N de Boysson H . ANCA and anti-MBG double-positive vasculitis: An update on the clinical and therapeutic specificities and comparison with the two eponymous vasculitis. Rev Med Interne. (2020) 41:21–6. doi: 10.1016/j.revmed.2019.10.334 31839271

[8] Asim M Akhtar M . Epidemiology, impact, and management strategies of anti-glomerular basement membrane disease. Int J Nephrol Renovasc Dis. (2022) 15:129–38. doi: 10.2147/ijnrd.s326427 35418771 PMC8999706

[9] Liu Y Wu Y Wei W Yang L Liu C Li J . Plasmapheresis, immunosuppressive therapy and anti-GBM disease prognosis: a cohort study of 107 patients. Ren Fail. (2024) 46:2400539. doi: 10.1080/0886022x.2024.2400539 39258391 PMC11391867

[10] Alchi B Griffiths M Sivalingam M Jayne D Farrington K . Predictors of renal and patient outcomes in anti-GBM disease: clinicopathologic analysis of a two-centre cohort. Nephrol Dial Transplant. (2015) 30:814–21. doi: 10.1093/ndt/gfu399 25609740

[11] Cui Z Zhao MH . Advances in human antiglomerular basement membrane disease. Nat Rev Nephrol. (2011) 7:697–705. doi: 10.1038/nrneph.2011.89 21769105

[12] Levy JB Turner AN Rees AJ Pusey CD . Long-term outcome of anti-glomerular basement membrane antibody disease treated with plasma exchange and immunosuppression. Ann Intern Med. (2001) 134:1033–42. doi: 10.7326/0003-4819-134-11-200106050-00009 11388816

[13] Connelly-Smith L Alquist CR Aqui NA Hofmann JC Klingel R Onwuemene OA . Guidelines on the use of therapeutic apheresis in clinical practice - evidence-based approach from the writing committee of the American Society for Apheresis: The ninth special issue. J Clin Apher. (2023) 38:77–278. doi: 10.1002/jca.22043 37017433

[14] Eckardt K-U Kasiske BL . KDIGO clinical practice guideline for glomerulonephritis2012. doi: 10.3238/arztebl.m2025.0082

[15] Rovin BH Adler SG Barratt J Bridoux F Burdge KA Chan TM . KDIGO 2021 clinical practice guideline for the management of glomerular diseases. Kidney Int. (2021) 100:S1–S276. doi: 10.1016/j.kint.2021.05.021 34556256

[16] Usui T Kai H Noguchi K Morito N Usui J Saito C . Effectiveness of plasmapheresis in a patient with anti-glomerular basement membrane antibody glomerulonephritis with advanced kidney dysfunction. Intern Med. (2017) 56:2475–9. doi: 10.2169/internalmedicine.8571-16 28824070 PMC5643177

